# Fate of nitrogen in agriculture and environment: agronomic, eco-physiological and molecular approaches to improve nitrogen use efficiency

**DOI:** 10.1186/s40659-020-00312-4

**Published:** 2020-10-16

**Authors:** Muhammad Anas, Fen Liao, Krishan K. Verma, Muhammad Aqeel Sarwar, Aamir Mahmood, Zhong-Liang Chen, Qiang Li, Xu-Peng Zeng, Yang Liu, Yang-Rui Li

**Affiliations:** 1 College of Agriculture, Guangxi University, Nanning, 530005 China; 2 Key Laboratory of Sugarcane Biotechnology and Genetic Improvement (Guangxi), Ministry of Agriculture/Guangxi Key Laboratory of Sugarcane Genetic Improvement, Sugarcane Research Institute, Guangxi Academy of Agricultural Sciences, Nanning, 530007 Guangxi China; 3 Crop Sciences Institute, National Agricultural Research Centre, Islamabad, Pakistan; 4 Guangxi Crop Genetic Improvement and Biotechnology Laboratory, Nanning, 530007 China

**Keywords:** Nitrogen use efficiency, Assimilation, Nitrate, Ammonium, Enzyme, Fertilizer

## Abstract

Nitrogen is the main limiting nutrient after carbon, hydrogen and oxygen for photosynthetic process, phyto-hormonal, proteomic changes and growth-development of plants to complete its lifecycle. Excessive and inefficient use of N fertilizer results in enhanced crop production costs and atmospheric pollution. Atmospheric nitrogen (71%) in the molecular form is not available for the plants. For world’s sustainable food production and atmospheric benefits, there is an urgent need to up-grade nitrogen use efficiency in agricultural farming system. The nitrogen use efficiency is the product of nitrogen uptake efficiency and nitrogen utilization efficiency, it varies from 30.2 to 53.2%. Nitrogen losses are too high, due to excess amount, low plant population, poor application methods etc., which can go up to 70% of total available nitrogen. These losses can be minimized up to 15–30% by adopting improved agronomic approaches such as optimal dosage of nitrogen, application of N by using canopy sensors, maintaining plant population, drip fertigation and legume based intercropping. A few transgenic studies have shown improvement in nitrogen uptake and even increase in biomass. Nitrate reductase, nitrite reductase, glutamine synthetase, glutamine oxoglutarate aminotransferase and asparagine synthetase enzyme have a great role in nitrogen metabolism. However, further studies on carbon–nitrogen metabolism and molecular changes at omic levels are required by using “whole genome sequencing technology” to improve nitrogen use efficiency. This review focus on nitrogen use efficiency that is the major concern of modern days to save economic resources without sacrificing farm yield as well as safety of global environment, i.e. greenhouse gas emissions, ammonium volatilization and nitrate leaching.

## Introduction

Nitrogen (N) plays an important role in crop plants. It is involved in various critical processes, such as growth, leaf area-expansion and biomass-yield production. Excess NUE can support good plant performance and better crop out-put. Various plant molecules such as amino acids, chlorophyll, nucleic acids, ATP and phyto-hormones, that contains nitrogen as a structural part, are necessary to complete the biological processes, involving carbon and nitrogen metabolisms, photosynthesis and protein production [1, 2]. Insufficient amount of N available to plants can hinder the growth and development. Nitrogen can also improve root growth, increase the volume, area, diameter, total and main root length, dry mass and subsequently increase nutrient uptake and enhance nutrient balance and dry mass production [3–6].

Application of nitrogen increases greenness of plants, CO_2_ assimilation rate, crop quality-yield and improve resistance to environmental stresses such as limited water availability and saline soil conditions [7, 8]. Hou et al. [9] found that nitrogen application more important than the other major essential fertilizers/nutrient for successful crop production. Consequently, N requirement is the most central feature for plant production [10]. Slow development of plant and early leaf senescence due to deficient N can cause decreased both crop production and quality [11]. Excessive N fertilizer application is common practice by farmers of cotton regions in the northwest [12] which is not cost effective for crop production, and excess N prolongs the vegetative growth period, delays maturity [13], decrease sugar content, and also attracts insect pest and causes disease epidemics.

**Fig. 1:**
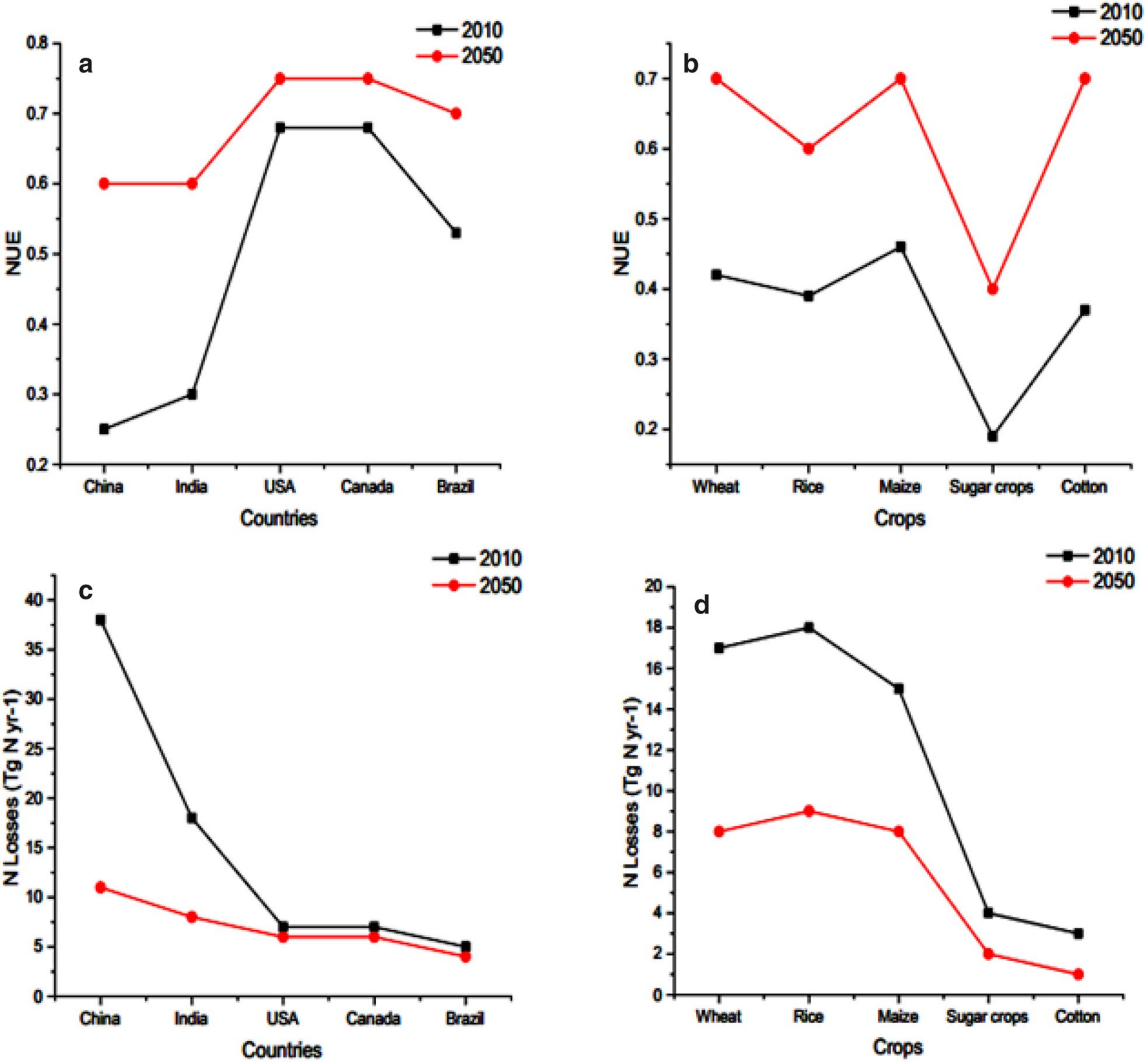
This diagram depicts country wise (**a**) and crop wise (**b**) NUE for 2010 and 2050 (proposed), while **c**, **d** shows nitrogen losses in teragram for 2010 and 2050 (proposed)

China has only 7% of global farm land with 20% world population that depends on it for feed [14–16]. It boosts up average yield of grain from 1.09 to 6.51 tonnes ha^−1^ in last 7 decades [17]. In China, chemical nitrogen (N) fertilizer input is the major element for the continuous increase of food production to mitigate the problem of food security [18]. Therefore, the low NUE all over the world especially in agriculture sector is not only wastage of resources (Fig. 1 a, b) and also problematic for environmental pollution (Fig. 1c, d) and conflicting to sustainable agricultural productivity [19–21].

## NUE and its status

**Fig. 2:**
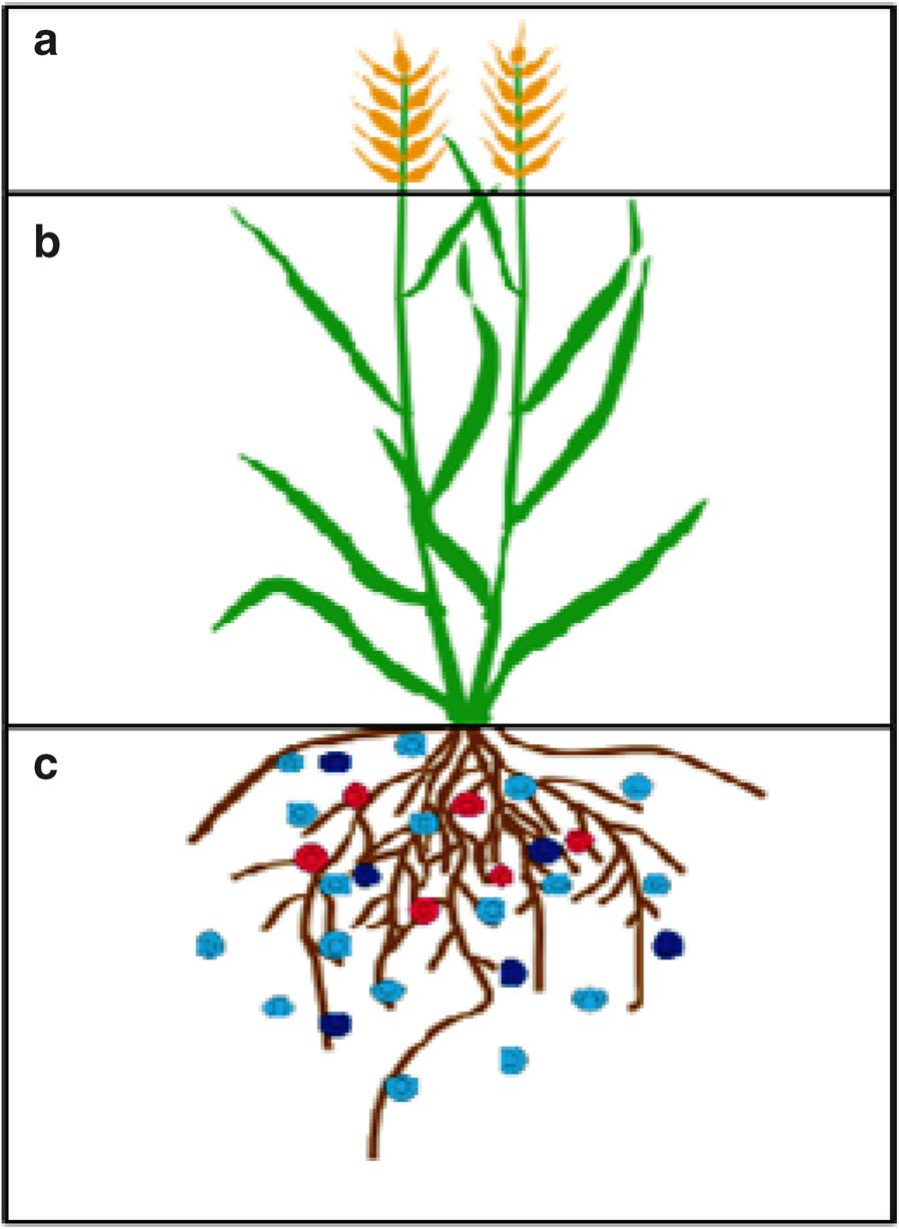
The major plant pats which have their own role for NUE. **a** Grain: responsive to fertilizers and nutrient storage component, **b** Shoot: nutrient redistribution, assimilation and transportation (source and sink), **c** Roots: Efficient nutrients uptake by transporters and channels

NUE is an exploiting issue for discussion and research which depends on the physiological and metabolic changes, such as soil nitrogen uptake, assimilation from roots to other parts (Fig. 2), source-sink tissues interaction for transportation, signaling and regulatory pathways which are responsible for N status within plant and growth as well [22]. Normally, the ratio of yield and total N supplied is termed into NUE [23]. Several techniques have been adopted to observe NUE that can be separated into N uptake efficiency and N utilization efficiency. N uptake efficiency (NUpE) describes the nitrogen amount that a plant can take from sources of nitrogen while N utilization efficiency (NUtE) termed as the plant capability to assimilate plus remobilize N within the plant [4, 22, 24]. However, NUE is the resultant of NUpE and NUtE product. Numerous demarcations for NUE have been suggested over the years, which have showed a few differences in normal ways [4, 25, 26].

NUE, NUpE and NUtE can be measured by adopting the Eqs. 1, 2 and 3 [4, 24].

1 $${\text{NUpE }} = {\text{ N contents in plant }}/{\text{N supplied}}$$

2 $${\text{NUtE }} = {\text{ Yield}}/{\text{N contents in plant}}$$

3 $${\text{NUE }} = {\text{ NUpE }} \times {\text{ NUtE}}$$

Nitrogen recovery and agronomic nitrogen efficiency (NRE) are the other common approaches used to observe NUE. NRE is termed as the percentage of pragmatic nitrogen fertilizer taken up by crop. It is an indicator for a crop to use the N fertilizer that has been supplied [27]. The yield increment per unit of N fertilizer given to the crop is denoted as agronomic nitrogen use efficiency (aNUE). It is an important index to measure gain or loss for excess amount of fertilizer [28]. Best aNUE is the surety of highest benefit–cost–ratio, which is a key economic relationship between input and output that relate both by linear curve [29].

The Eqs. 4 and 5 can be used to measure agronomic and recovery efficiencies like aNUE and NRE:

4 $${\text{aNUE }} = \, ({\text{Y}}_{\text{fertilized}} {-}{\text{ Y}}_{\text{not fertilized}} )/{\text{F}}_{\text{applied}}$$

Y_fertilized_ and Y_not fertilized_ are yields (kg ha^−1^) when quantity of N fertilizer applied was F and zero; F_applied_ is the total N (kg ha^−1^) applied [28].

5 $${\text{NRE }} = \, ({\text{Total NU}}_{\text{fertilized}} {-}{\text{ Total NU}}_{\text{not fertilized}} )/{\text{N fertilizer dose}}$$

Total NU_fertilized_ and Total NU_not fertilized_ showed N uptake for F and no fertilizer, respectively [30].

The variation in NUE can be understood by nitrogen doses, application methods and other agronomic factors which help to manage nitrogen has crucial effect for both profitable crop production and environment [31]. According to field demonstrations, Lou et al., [32] measured NRE and aNUE for different nitrogen rates, application methods and plant population in northwest, China, and found that the 70% and 80% of nitrogen loss can be minimized when nitrogen applied through drip fertigation and high plant population, respectively. Drip fertigation and high plant density can increase nitrogen recover efficiency for comparable yield. In contrast conventional method of nitrogen application and low plant population, more nitrogen losses, which leads to decrease yield in crops due to low amount of N available. The midseason rice NUE is less than 30% in China, which indicates that 70% nitrogen is going into the ecosystem as loss [33]. As comparison of USA and China from 1980 to 2010 for NUE in case of maize crop, the NUE declined from 30.2 to 29.9 in China but up-graded from 39.4 to 53.2 in USA [34]. Hajari et al. [35] demonstrated few varieties of sugarcane for nitrate and ammonium as a source of N fertilizer in their study and concluded that NO_3_^−^-N resulted in higher NUEs as compared to NH_4_^+^-N. Wheat and maize grown in a hydroponic culture containing NH_4_^+^-N showed that the photosynthetic and carbon assimilation rates decreased in the plants [35, 36].

### Available sources and forms nitrogen

The conversion of nitrogen from one form to others greatly influences the nitrogen use efficiency.In early growth stage NO_3_^−^ form of nitrogen is important but it has not been commonly used as fertilizers alone, the other forms go the atmosphere by nitrification [37]. However, most widely used nitrogen fertilizer urea is abruptly nitrified (Fig. 4) after conversion to ammonium [37]. Although urea after application in soil can convert into nitrate and ammonium form, it is not still clear about urea uptake process and metabolic changes in plants [38]. Urea is also preferred and predominant source of N due to more nitrogen contents and low cost to produce it in South Africa [39].

**Fig. 3:**
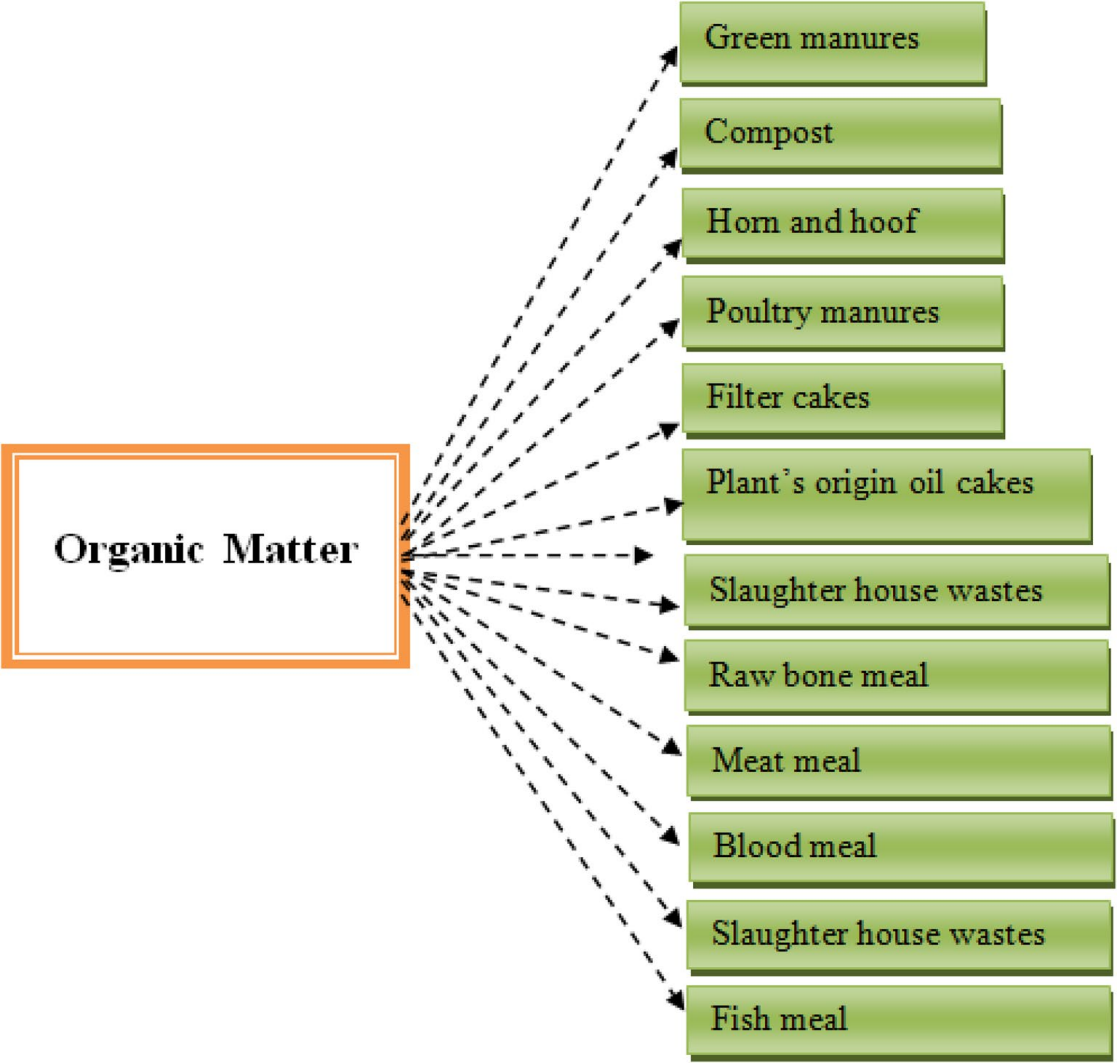
Sources of organic nitrogen available for mineralization in soil [59]

The soil N (Fig. 3) is most important to observe the efficiency of N in the agricultural field conditions [40–44]. There are a lot of evidence from various field trials using ^15^N-labeled fertilizer, N uptake is principally derived from soil (Fig. 3) rather than fertilizer [45–53]. However, many studies have been conducted and found that unfertilized N responses often give more yield than that of N fertilized [43, 54–56], except those in which soil N availability is captured by accumulation of carbonaceous residues. Total soil nitrogen and organic carbon vary in soil profile, both decreases with the soil depth, however the ionic forms of N (NH_4_^+^, NO_2_^−^, and NO_3_^−^) shape the mineral nitrogen dynamics because discrepant increments of mineral nitrogen stock in each soil layer takes place [57, 58].

According to Neto et al. [60] when nitrogen concentration increases even though it is earlier applied, mineralization of nitrogen in soil is boosted and a part of N shares from the mineralized nitrogen. Nitrogen within the plants at anthesis stage also enhanced due to the transformation of nitrogenous compounds, which have stored nitrogen in earlier growth period [61, 62]. Crop growth, development, biomass and yield have directly linked to nitrogen assimilation [61, 63]. Mazzafera and Goncalves [64] analyzed xylem sap to study nitrogen transformation in coffee plants and found 52% of the total nitrogen is nitrate. But nitrate reductase reduces it into nitrite [65].

Sugarcane accumulates nitrogen 100–150 kg ha^−1^ in leaves and stalks, only about 55% is removed from stalks up to maturity [66]. The plant residues after harvesting are put into the field which gradually mineralized and release N in available forms [67]. Nitrogen consumption by enhanced N fertilization to the crop may lead to high N uptake but it is not necessary to increase biomass production [68]. Thus, over use of nitrogen fertilizer down-regulates the nitrogen use efficiency and increases production cost and environmental pollution.

Plants have the ability to acquire excessive NO_3_^−^ nitrogen than the requirement for assimilation and store it in unassimilated pools like vacuoles of leaves [69], become available for utilization under low N [70, 71]. Hajari et al. [35] and Robinson et al. [37] found, the NO_3_^−^-N per gram was higher in dry roots than the shoot on all growing media. Hajari et al. [35] claimed that the sugarcane plant is not able to translocate NO_3_^−^-N from root to shoot efficiently due to which limited N uptake and transport occur rather than assimilation which may affect the NUE in sugarcane. The application of a nutrient may increase (synergism) or decrease (antagonism) the contribution of the other nutrients in crop yield. The concentration of phosphorus and nitrogen varies over the growing period in the soil and create interaction either synergistic or antagonistic. The response of crop yield might be affected directly or indirectly [72, 73]. Therefore, the supply of both N and P creat changes in chemical, physical, and biological properties of soil [74, 75]. The nitrogen fertilizer has synergetic effect to phosphorus. The results indicated, addition of nitrogen along with phosphorus fertilizer produced better positive interaction than separately [76]. In the sugarcane field which has previously wild vegetation and low available phosphorus response nutrient limitations, it involves phosphorus as limiting source in high demand periods, and also microbial biomass [77–85].

### Losses of nitrogen in the ecosystem

**Fig. 4:**
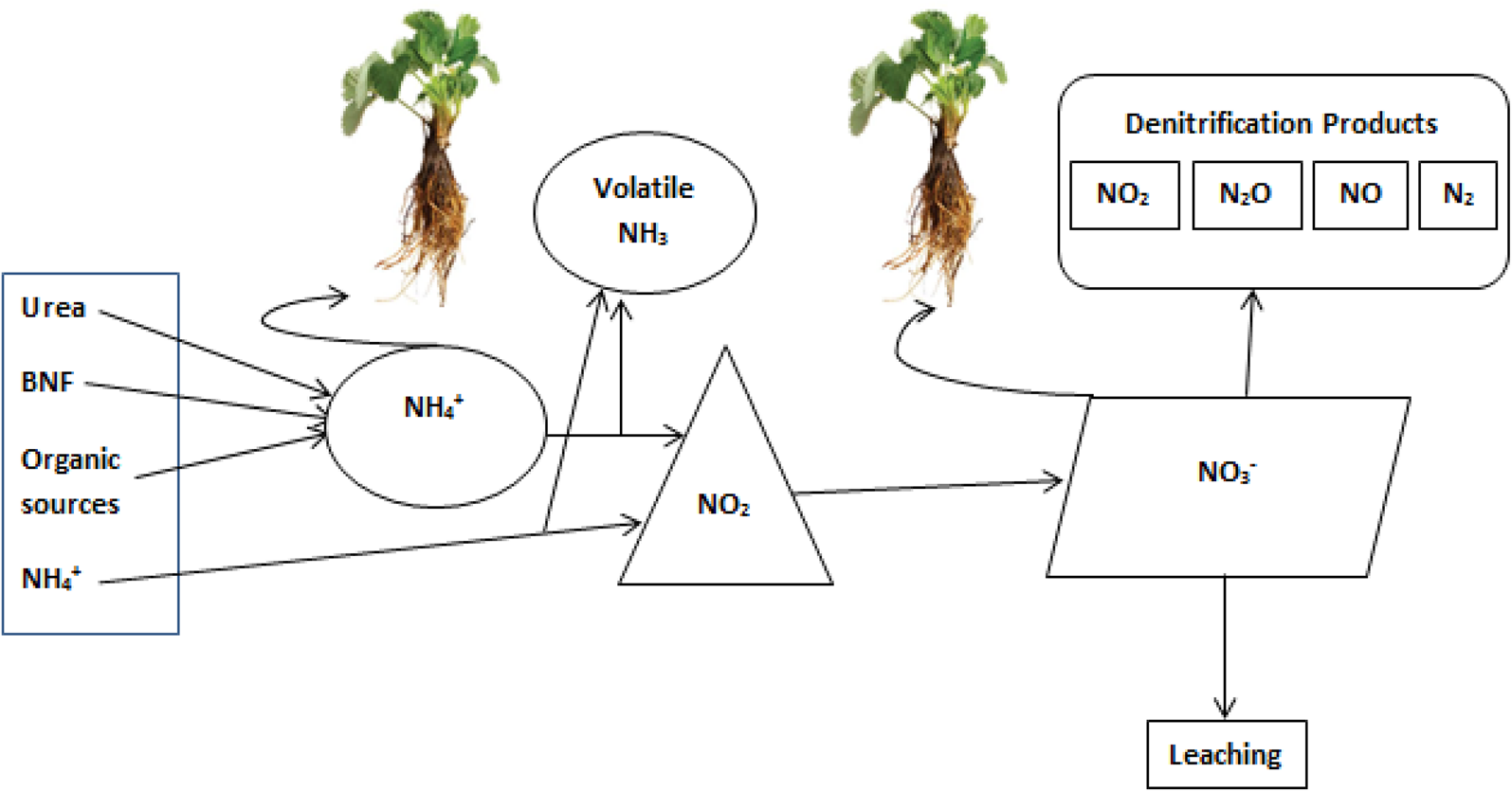
Summary of nitrogen sources and, their conversion, availability to plants and losses within/outside of soil

Worldwide high nitrogen fertilizer application results in economic loss and ecological hazardous due to extra consumption of resources, water eutrophication, and high rate of greenhouse gas emissions along with potential leaching. The inefficient N utilization with poor transformation of provided N results in unintentional fertilizer loss in soil, atmosphere and promoting contamination of groundwater, distort connecting biological communities and cause dangerous atmospheric deviation, through the emission of the poisonous ozone depleting substance nitrous oxide [82], eutrophication, air pollution, N leaching, water pollution, soil acidification and soil degradation [14, 18, 82–89] which is not suitable for environment friendly crop production and human life (Fig. 4).

In agriculture, crop production requires plentiful N which is the most widely recognized limiting factor for crop growth, development and yield. A lot of synthetic N fertilizer is applied to arable land by growers to fulfill the demand for crop production. An abrupt increment in fertilizer applications in China was noted, and it consumed 30% of total N fertilizers synthesized around the world in 2002, in spite of the facts, its arable land accounts only 10% of the world aggregate. However, the use of vast amounts of synthetic N fertilizer to expand crop yield are not financially sustainable and put a substantial burden on farmers, and furthermore result in environmental pollution. Every crop cannot use about 50% nitrogen fertilizer during its growing season due to over fertilization [90].Moreover, plants grown under excessive nitrogen applications are more susceptible to lodging because of shoot overgrowth and tender, and pest damage and disease, and also degrade quality of the grains [91].

The N losses thru lixiviation, direct escape to the air, denitrification and/or percolation is higher due to over use of N fertilizer [92]. The synchronized application as the demand of plant at its critical stage can decrease losses of applied N fertilizer [93–95]. Over the last decade, crop response to N fertilization [96, 97] was detected in sugarcane fields all over the Brazil for green cane trash blanketing systems (GCTBS) and also in situ quantify NH_3_ volatilization [98], NO_3_ leaching [99–101], and N_2_O emissions [102, 103], N use efficiency [104, 105]. About 60–80% synthetic N fertilizer is not taken up by sugarcane crop under GCTBS, and losses due to volatilization, denitrification and leaching has been observed, but most of the mineral N is not available for micro biota, while the remaining part available to the crop [96]. In spite of the fact that the mechanism of commercial fertilizers is relatively well familiar [106]. However, many researchers claim the impact of organic and organomineral is not understood on chemical and microbial properties of soil for successful crop cultivation in temperate areas [107–109].

Biotic factors like size and diversity of microbial community and abiotic factors temperature, soil moisture content, temperature have direct relation to regulate organic compounds mineralization in the soil (Fig. 3), however, seasonal climatic change during cropping season fluctuate the mineral N availability [110]. Rapid availability of mineral N in soil solution has been noted as a result of synthetic N fertilizer application [96, 111, 112], but there is a powerful race between crop plants and micro flora for existing mineral N (especially NH_4_^+^), and cause a large variations over time [77, 78].

Urea is the major N fertilizer that is applied to the field and also the main source of NH_3_ gas emission (Fig. 4) from agronomic practices [113] contributing for about 20% of the emissions in Germany [114] and is highly important in many other countries like China. Nitrogen loss as NO_3_^−^ leaching (Fig. 4) from sugarcane field has significant contribution to pollute environment in Australia [115]. Many researchers in Brazil also find out leaching losses of nitrogen in planted sugarcane throughout its growth [116]. However, during ratoon season, NO_3_^−^ leaching is more important than the planted cane [100]. The skips within ratoon sugarcane field increased across the growth period, and decreased the crop N response. The unique response to applied N fertilizer can be attained by well-established ratoon crop similar to planted crop density.

Duan et al. [117] discuss their findings about N application to long and short vines of sweet potato, the both long-vine and short-vine cultivars have the peak yield for nitrogen applied as 30 and 90 kg ha^−1^ respectively. The cultivars of same production potential have reduced their yields, and the root yield of long vine is significantly lower than that of short vine for nitrogen 120 kg ha^−1^. Wu et al. [118] also claim the cultivar Zijing No. 2 decrease in the root yield for N application (75 kg ha^−1^) in fertile soil. Thus, the genotypic differences in sweet potato have a great influence on the partitioning of dry matter as well as uptake of nitrogen [119]. Wilson [120] classified cultivars of sweet potato for N-responsiveness, nonresponsive and depressive natures. Nitrogen buildup and distribution for short stature tuber roots are greater, and similarly exhibit more yield in response to high N conditions [121]. Besides, the cultivars that require higher N, give higher root yield in fertile soils [118].

Total nitrogen fertilizer can be reduced up to 360 kg ha^−1^ with respect to 430 kg ha^−1^ for cropping system based on the wheat–maize rotations, along with improved agronomic practices. It was resulted in increase in maize yield by 7–14%, but reduction in wheat yield, N_2_O and NO emissions by 1–2%, 7% and 29%, respectively [122]. In addition, best fertilization practices are an option to improve NUE and also seasonal collective N_2_O emission decrease [123]. Leaching process can be minimized by adopting legume crops in cropping system up to 50% than the conservative systems [124]. Soybean reduces 50–60% of N demand by biological nitrogen fixation [125]. Graham et al. [126] and Resende et al. [127] observed that addition of synthetic fertilizers decreased soil N stocks, while Ladha et al. [108] reported an increase in soil C pool and N stocks for long–term organic fertilizer application.

### Agronomic and physiological approaches

#### Application rates

Irrational application of nitrogen is a major problem of low nitrogen use efficiency [128–130]. Therefore, agronomic principles and practices should utilized in modern techniques to enhance nitrogen use efficiency, so as the reduced application rate of fertilizer inputs without yield reduction is key factor [32]. Soil characteristics and agro-climatic conditions highly force the application level of fertilizer [131]. Crops can use only up to 35% of the supplied N during its complete life cycle [39] and the remaining is escaped to the environment by various mechanisms and functions (Fig. 4) [132, 133].

Improvements in NUE by decreasing nitrogen dose may delay leaf senescence which results in no yield loss. Late-season leaf senescence due to low nitrogen application rate provides relatively higher photosynthetic capacity to crop and ultimately increase yield production. Mulvaney et al. [109] proposed N mineralization in soil is positively regulated by synthetic nitrogen fertilizer. These findings indicate that N may exceeds the demand of sugarcane crop (200 kg ha^−1^ year^−1^) and affect C:N ratio in soil for long time continuous applications.

Srivastava and Suarez [134] confirmed N recommendation rate for sugarcane varies worldwide for 45 to 300 kg ha^−1^ but 60 to 140 kg ha^−1^ is recommended for Brazil. Dametie and Fantaye [135] summarised the results of sugarcane N uptake studies by various researchers in the globe, and indicated that the usual need of ratoon crop for nitrogen is 1.5 kg Mg^−1^ cane yield. N uptake varied from 0.88 to 1.47 kg Mg^−1^ in Hawaii, and stubble cane production required 1.3 kg Mg^−1^ [136, 137]. By the compilation of numerous results for nitrogen dosage and technically recommendations in Brazil, the usual rate is 1.0–1.4 kg Mg^−1^ cane [138].

Nitrogen fertilizer application dose can be minimized by 20% without yield loss in Australia [139]. The N fertilizer in China has possibility to use moderately at low rate by integration management practices [140]. The reports from different regions/countries suggest that N use efficiency can increased by decreasing N application rate [141–144]. However, it also depends on agronomic traits, fertility of soil, management and yield potential [141–144].

The N application rate can also be determined by vegetative growth and productivity index, for example, coffee plants showed high rates for it between 2400 and 3600 kg ha^−1^ per year [60, 145] and N as urea applied 600 to 800 kg ha^−1^ to maintain this productivity in Brazil. Official recommendations for nitrogen fertilizer are 400 kg ha^−1^ year^−1^ [61] and apply in tow or four splits. But the coffee growers applied urea between 600 and 800 kg ha^−1^ in 26 splits during coffee cycle. In fact, they attempted this practice to stop N deficiency, but causing low nitrogen use efficiency [146]. Luo et al. [32] suggests that 20% N can be reduced, when plant density is high, without yield loss and also can reduce for drip fertigation.

#### Application methods

The international plant nutrition institute is convincing the best agronomic practices, 4R nutrient application principles, i.e. source of fertilizer, rate, time and site/place [147]. Soil fertility varies with in the field abruptly which has strong impact on yield and nutrient uptake by cultivated crops, and this major problem can be handled by adopting site-specific nitrogen fertilization. Site-specific N fertilization provides significant impacts in terms of economy and ecology in heterogeneous fields [148–150] which results in enhanced yield, quality and ultimately high nitrogen use efficiency.

Spectral measurement is a suitable approach to know the nitrogen requirements of crops and site-specific application for precise farming [151]. The principle behind laser-induced chlorophyll fluorescence (LICF) is used to the measure the N situation of the crop stand by close distance [152] as well as 3–4 m [153]. The plant nitrogen is measured indirectly by chlorophyll content via fluorescence signals ratio at 690 and 730 nm [154, 155]. It indicates that high amount of chlorophyll resulted in lower fluorescence radiation ratio F690/F730 because reabsorbed radiations have more strength at 690 nm. Rubisco acts as the sink of N and has close relation to chlorophyll content, thus the ratio F690/F730 describes the N content of the plant [156].

Crop canopy sensor calibration is too sensitive to field variability like the ramp calibration strip [157] or the calibration plot methods [158]. The reference area for canopy sensor within a field should be given according to field and soil variability [159] that also relates to the sugarcane plant density variation. The calibration should be done for every crop and season, separately [160]. Yong et al. [161] applied nitrogen fertilizer at various concentrations among the rows of maize-soybean relay intercropped field at three different distances (15 cm, 30 cm and 45 cm) and concluded that crop performed better for 15 cm and 30 cm treatments. The NUE and total grain yield of the maize-soybean relay intercropping system were significantly higher in 15 cm and 30 cm. So, lower N application at 15–30 cm from fertilizer application location to the maize row was optimal.

Productivity of low land rice has a great dependence on the selection of varieties and their nutrient utilization capacity. Under dose of N fertilizer may happen, especially when N is subject to immobilization following ratoon crop fertilization for unburned sites [56]. Crop response to inputs is also influenced by climate, for example, high altitude of Andhra Pradesh is endowed with the special soil and climate where varietal responses to inputs vary relatively to coastal plains. Different nitrogen sources should be jointly applied to fulfill the requirement of nitrogen to improve crop productivity [162].

The supply of N fertilizer to sugarcane is affected by soil profiles that are hard to measure inside the agricultural land [53]. Indeed, even the selection of reference regions, that get satisfactory measures for nitrogen, according to Raun et al. [163], can be risky with regards to evaluating sugarcane N feedback; depending upon where reference zones were set up, the harvest N reaction can differ altogether. For instance, producers may realize that a yield did or did not respond to N application,and such conflicting results found in various experiments were demonstrated by Duan et al. [117]. Hence, use of canopy sensors to quantify the N response is troublesome because of variable plant density inside the fields. In that capacity, different elements can veil the N impacts, like soil compaction, pest attack and diseases. Zillmann et al. [164] announced a comparative issue when they conducted a test for N connected to maize. For all the experimental area, the crop response for N was not similar as proposed.

The canopy sensor has to be utilized when the sugarcane tallness is between 40 and 70 cm to get estimation affectability to sugarcane vigor fluctuation [165, 166]. At this stage, sugarcane has attained around 10–30% of total biomass with 27–68% N, which is dependent on genotype, soil fertility, climate and developmental stage [167]. N requirement of crop prior to treatment can achieved by various sources, i.e. mineralization of organic sources and endophytic nitrogen fixation by bacteria related to plant roots [53, 168–170], and also other inputs to the field like vinasse, poultry manure and farmyard manure etc.

## Drip fertigation

Northwestern China has an arid climate, cotton production in this region is not possible without irrigation and N fertilization [171]. Drip fertigation is a good option to supply water and fertilizers in precise quantities [172, 173]. Drip fertigation with mulching is going to be extensively used in recent years [174]. It is well documented that the nutrient and water use efficiency both can be enhanced through drip fertigation that improves crop production for each unit of nutrients and water [172, 175]. It has more advantage of the soluble fertilizers that can be put in specific quantity alongside the good crop health and potential yield because of maintained fertigation in the root zone [173]. Many studies pointed out fertigation can improve fertilizer use efficiency by decreasing application rates without losing crop yield [176, 177] and especially drip fertigation of cotton field with reduced nitrogen, improved its efficiency [175, 178]. It improved cotton yield, yield components, and leaf area index (LAI) by 20 to 30% as compared to furrow irrigation [179]. However, maximum nitrogen recovery was obtained by sacrificing cotton yield at lower N level under drip fertigation [180]. So, an optimum N level for drip fertigation has important role to achieve highest cotton yield.

Traditional high nitrogen application without considering method of application and plant population gives more seed cotton yield. Anyhow, N can be reduced up to 15–30% when drip fertigation is employed and 20% in case of high plant population without sacrificing seed cotton yield. The findings of Luo et al. [32] are that N reduction up to 30% has non-significant seedcotton yield reduction for drip fertigation. However, drip fertigation shows increase by 5 and 20.7% in seedcotton yield for 15 and 30% nitrogen reduction.

In other words, drip fertigation with high plant population is an important attribute to save nitrogen with sustainable yield for arid culture. Many experiments have conducted to find agronomic practices, high planting density, diversified planting geometry [181] organic fertilizers and improvement of application method of nutrients are helpful to regulate cotton yield for reduced nitrogen conditions in the Yellow River valley, China [11, 12, 140].

## N and plant density

The plant density is an important tool to testify N rate without sacrifice of yield either by increase or decrease in number of plants per unit area [12, 140, 182]. It varies active crop canopy reflectance on the base of ground for sensors [183]. This idea has been proficiently utilized to control N application for rice [121], maize [184–188], cotton [189] and wheat [188, 190, 191]. The application of nitrogen based on canopy sensor depends on chlorophyll of crop canopy which describes nitrogen status [192], but it is not as valid for sugarcane. The field-scale sensor observations at the leaf level poorly show a relationship with nitrogen and chlorophyll status [166]. It is due to irregular sugarcane canopy which may show ground soil to the sensor. Dynamic and manually monitored canopy reflectance sensors are available, which consider all the parameters for sugarcane biomass variation, principally effected by plant population, as described by Amaral et al. [138].

Amaral et al. [138] conducted strip experiments for different nitrogen rates and validated that the uniform distribution of canopy has no trouble for canopy sensor. Variation in the canopy is mainly affected by plant population and vigor rather than the nitrogen supply. Six trials with differing nitrogen supply were conducted at different locations, five out of six trials has non-significant response to variable nitrogen supply and the sixth trial may have variation in soil characters, deeper root zone and more water holding capacity, therefore increases soil nutrient utilization and crop vigor.

## Intercropping

Intercropped crops are significantly influenced by fertilization methods and show better growth for diverse nitrogen supply for interspecific rows instead of intraspecific [193]. Interspecific applications accelerate resource use efficiency, soil productivity and also have positive impacts on the environment [194–197]. This system involves more than one crop in a season, and can be observed in the Huang Huai Hai, China [198], and relay intercropping system is common in the Southwest China where one crop or three crops in 2 years are grown [199]. So, better nitrogen fertilization methods and relay or intercropping systems based on soybean (legume crop) greatly influenced on soybean yield with decreasing environmental cost. But environmental features like rainfall, light intensity and heat can be limiting factors for cropping systems. Maize-soybean relay intercropping occupies largest planting area in Southwest China that is helpful to improve nitrogen, light use efficiencies and soil nutrient availability [20, 199–204].

There are many previous studies indicating that high N input has undesirable outcome for biological nitrogen fixation [205]. When nitrogen availability studied for legume-nonlegume mixtures, high content of mineral nitrogen in soil triggers the microbial nitrogen fixation and hence availability of nitrogen decrease for nonlegume crop [206]. However, low input of nitrogen increased significantly fixation and stimulated the translocation of fixed N to nonlegume [203, 207].

## NUE regulating enzymes and genes

The major sources of nitrogen, taken up by higher plants, are nitrate and ammonium as synthetic fertilizers, organic compounds and amino acids etc. It depends upon the availability of nitrogen, and within the plants it is controlled by many metabolic pathways and genes expression levels [208]. Nitrogen use efficiency is dependent of soil nitrogen conditions, photo synthetically fixed carbon dioxide to provide precursor for biosynthesis of many amino acids and vice versa [209, 210]. It has been also claimed that all the inorganic nitrogenous fertilizers first converted to ammonium before uptake by higher plants [211]. Nitrate reduction occurs in roots as well as shoots but nitrate reduced directly in cytoplasm while in plastids/chloroplast via nitrite [208]. Reduction of nitrate to nitrite occurs in cytosol by nitrate reductase enzyme (Table 1) [212]. Nitrite is transported into chloroplasts in leaves where nitrite is converted to ammonium ions due to nitrite reductase (Table 1) [213]. The products of ammonia, glutamine and glutamate, act as donor of the nitrogen during biosynthesis for nucleic acid, chlorophyll and amino acids. The isoenzymes of glutamine synthetase, glutamate synthase, and glutamate dehydrogenase (Table 1) have been proposed for three major ammonium assimilation processes: primary nitrogen assimilation, reassimilation of photorespiratory ammonia, and “recycled” nitrogen [213]. Organic nitrogen in the form of amino acids transferred from source organs to sink (Fig. 2), for example, glutamine and glutamate can be used to form aspartate and asparagine [211, 214]. The ammonium nitrogen is transferred into amino acids by the enzymes e.g. glutamine synthetase, glutamate synthase, asparagine synthetase and aspartate amino transferase (Table 1). The coherent situation existed for glutamate dehydrogenase either it is involved in assimilation of ammonium nitrogen or carbon cycling [215, 216].

**Table 1:** The basic information of enzymes involved in nitrogen metabolism of plants

Enzyme	Abbreviation	Encoding genes	Function
Nitrate reductase	NR	5	Reduce nitrate ion into nitrite ion
Nitrite reductase	NiR	30	Further reduce nitrite into ammonium ion
Glutamine synthetase	GS	49	Involve in GOGAT pathway
Glutamine oxoglutarate aminotransferase	GOGAT	15	Involve in GOGAT pathway
Glutamate dehydrogenase	GDH	3	Dehydrogenate α-ketoglutarate
Aspartate aminotransferase	AST	13	Catabolise glutamate into aspartate
Asparagine synthetase	AS	4	Aspartate is converted into asparagine

**Fig. 5:**
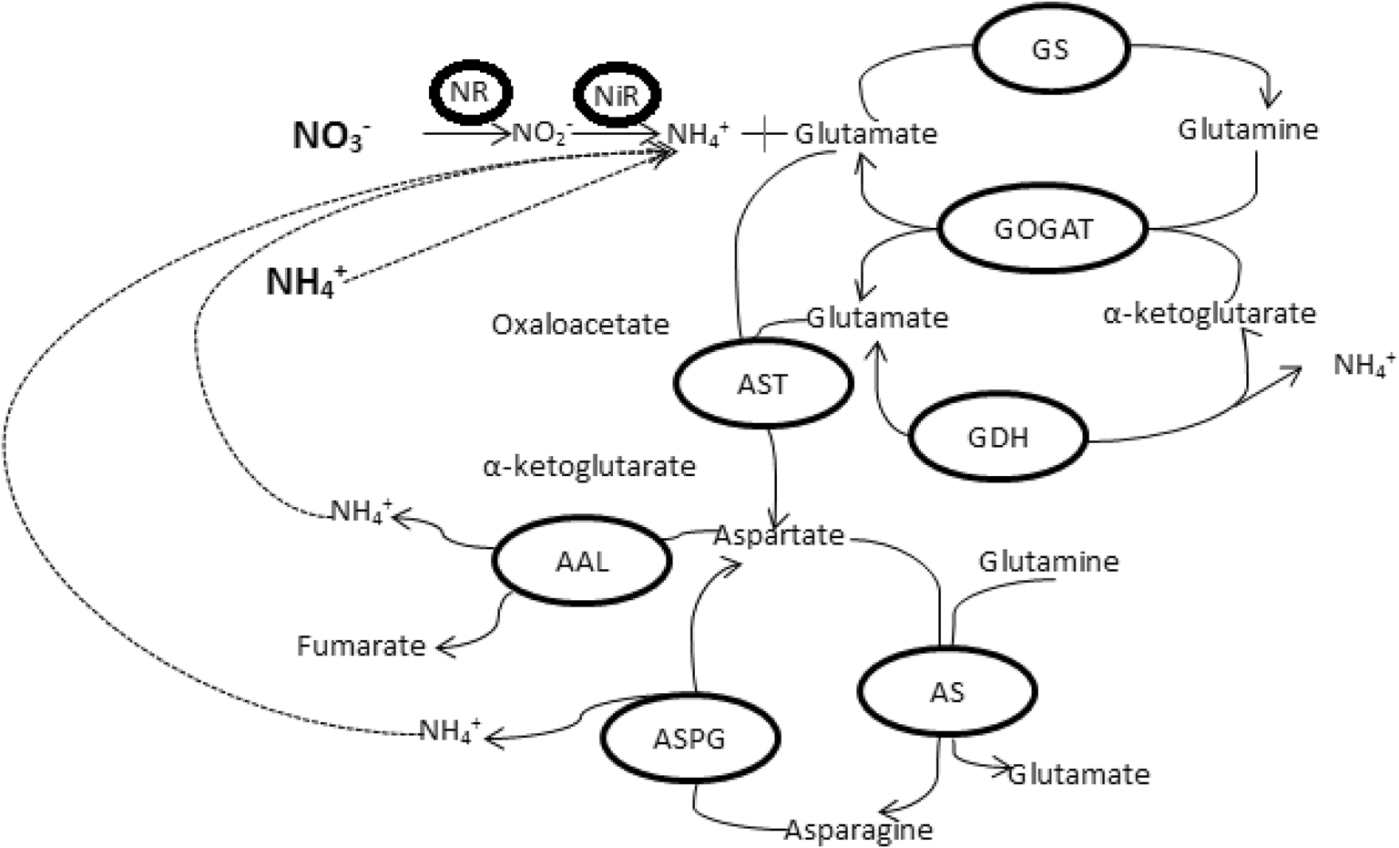
Schematic diagram to show the fate of nitrogen within the plant Bolded **NO**_**3**_^**−**^ and **NH**_**4**_^**+**^ are nitrogen uptake forms by roots through different transporters

The ammonium assimilating enzymes are important during grain filling stage due to its remobilization. The biosynthesis of amino acids from ammonia is occurred by the GS and GOGAT pathways (Fig. 5) [217]. Nitrogen reutilization is an important phenomenon involving NADH-GOGAT enzyme, rice grain weight increased up to 80% due to over production of NADH-GOGAT [218]. Glutamine dehydrogenase involves for senescing of leaves and also controversy as deaminating (Fig. 5) [219, 220] and aminating directions [23]. Young leaves recycle nitrogen from chloroplast by GS2 and Fd-GOGAT. In GOGAT catalyzed proteolysis, GS2 and de facto NiR are responsible for breakdown of chloroplast during senescence. Production of glutamine during leaf senescence is basically dependent on GS1 isoform. Substrates for GDH are produced from chloroplast proteins proteolysis, and deaminating activity provides 2-oxoglutarate and ammonia. Glutamine for new sink organ is produced by GS1 reassimilation of ammonia [221].

Each monomer of homodimer nitrate reductase associated with three prosthetic groups: flavin adenine dinucleotide (FAD), a molybdenum cofactor (MoCo) and a haem. NR reduces chlorate into toxic chlorite, responsible gene for that in mutant has been identified, the *Nia* genes encoding the NR apoenzyme and the Cnx genes encoding the MoCo cofactor. [208, 222]. The *Nii* genes have one to two copies encoding the NiR enzyme [208]. GS having decameric structure is controlled by two classes of genes, *GLN1* and *GLN2*, [223]. *GLN2* (single nuclear gene) encodes chloroplastic GS2, involved in ammonium assimilation or re-assimilation either from nitrate reduction in C_3_ and C_4_ plants or photorespiratory product of C_3_ plants [224]. On the other hand, GS1 isoform is encoded by *GLN1* gene family which recycles ammonium during leaf senescing and transport in the phloem sap [225]. Vanoni et al. [226] reported that GOGAT (mechanistic structure) has two forms Fd GOGAT (in leaf chloroplast) and NADH GOGAT (in plastids of non-photosynthetic tissues). Three genes (*ASN1, ASN2* and *ASN3*) encode asparagine synthase, and substrate ammonia is utilized by asparagine synthase to form asparagine [227]. Storage compounds, long-range transporter and glutamine has lower N/C ratio than asparagine [228, 229]. In plastids; bicarbonate, adenosine tri-phosphate and amide/ammonium from glutamine act as substrate for carbamoylphosphate synthase (CPSase) to form precursor (carbamoylphosphate) of citrulline and arginine. The subunits (small and large) of carbamoylphosphate synthase (CPSase) encoded by *car A* and *B* genes, respectively [230]. Finally, glutamate is produced by mitochondrial NADH-glutamate dehydrogenase for higher levels of ammonium [23].

### NUE responsive genes manipulation

Crop varieties that are highly N efficient, high yields with reduced N input is the main solution for improving NUE [231–233]. Recent studies documented that shoot-to-root signaling pathways, feedback mechanisms and amino acids transportation in roots and shoots influence the nitrogen uptake and its metabolism [234–238]. With the aim of improving NUE, approaches have been adopted on the basis of genetic changes for nitrogen uptake [239–241], nitrate allocation [242], nitrogen metabolism [218, 243–249] and the regulation [250].

Many critical candidate genes also have been over-expressed and knocked out in order to test for biomass and plant nitrogen status. Nitrate influx increased due to over-expression of HATS-like NRT2.1 but at the same time NUE and its utilization phenotypically remains unchanged [46]. Overexpression of genes encoding for NR/NiR in transgenic plants to improve NUE has no surety for its utility. Nitrate reductase related gene overexpression in tobacco plants showed delayed NR-activity for drought conditions and quick recovery for re-watering after short time drought [251]. It has been observed that nitrate level decreased in transgenic Arabidopsis, tobacco and potato plants without improving in biomass, number of tubers and seeds respectively. Regardless of the nitrogen available sources, *Nia* or *Nii* genes overexpression improved mRNA levels besides N uptake affect without any change in the yield and growth, indicating the composite post-transcriptional regulation of NR [252].

When we talk about *GS1* and *GS2* genes expression, the overexpressed *GS2* has been testified along with Rubisco promoter in *Nicotiana tabaccum* and CaMV 35S promoter in *Oryza sativa* [4, 217]. It enhanced growth rate in *Nicotiana tabaccum* and photorespiration and drought tolerance in *Oryza sativa*. Overexpression of *GS1* genes with promoters having different combinations, RolD, CaMV 35S and Rubisco subunit (rbcS) have been reported with positive results for plant biomass and grain yield. For example, grain yield and roots are significantly higher with more N content in nitrogen efficient wheat lines under the control of the rbcS promoter observed [248]. Similarly, biomass and leaf protein in *Nicotiana tabacum* (over expressed *GS1*) increased under the control of CaMV 35S promoter [253]. Another overexpression of *GS1* gene depicted 30% increase in yield of maize due to more kernel number and size [231]. In conclusion, GS activity has direct relation with biomass or yield in transgenic plants [254]. Over-expression of NADH-GOGAT increased in grain yield for transgenic rice plant [231]. So, it is important to know the alleles of genes and promoters to improve yield by overexpressing GS or GOGAT genes. Overexpressed ASN1 in Arabidopsis increased soluble protein content in seed, total protein and plants ability to grow for limited nitrogen supply [229]. These results suggested that NUE can be improved by manipulating downstream steps in N-remobilization. Further studies of carbon metabolism pathways also have potential to improve NUE [255–257].

Several external and endogenous factors influenced the expression of genes which are highly regulated at the transcriptional as well as post-translational levels [208]. Lea et al. [218] demonstrated that post-translational regulation affects the amino acids, ammonium, and nitrate levels, whereas transcriptional regulation has only minor influence. Plants unregulated for NR accumulate high concentrations of asparagine and glutamine in leaves. Thus further characterization can provide the useful properties for crops.

Asparagine synthetase (AS) encoded by a small gene family, catalyzes the formation of asparagine (Asn) (Fig. 5) and glutamate from glutamine (Gln) and aspartate [258]. The role of AS and GS interaction in primary N metabolism is very crucial [259, 260]. GS negatively correlates with the AS transcript levels and polypeptides in the transgenic plants suggesting that AS showed compensation for GS ammonium assimilatory activity [260, 261]. It is hypothized that AS might be important in regulation of the reduced N flux into plants due to decreased GS activity. However, the GS is essential to synthesize Gln for biosynthesis of Asp via NADH-GOGAT and AspAT [260]. Lam et al. [229] demonstrated the results of overexpressed the *ASN1* gene in Arabidopsis as enhanced soluble seed protein content, total protein content with better growth on N-limiting medium. However, in case of *ASN2* gene endogenous ammonium accumulation was less compared to wild-type plants as growing on 50-mM ammonium medium [22]. Signaling processes are attractive clues for metabolic engineering. Physiological activity of glutamate dehydrogenase (GDH) is still unclear as compared to GS/GOGAT enzymes [215]. Ameziane et al. [241] investigated GDH activity in transgenic tobacco plant, and the biomass production increased in *gdhA* transgenic plants without considering growing conditions either controlled conditions or field.

### Microarray and whole genome sequencing

**Fig. 6:**
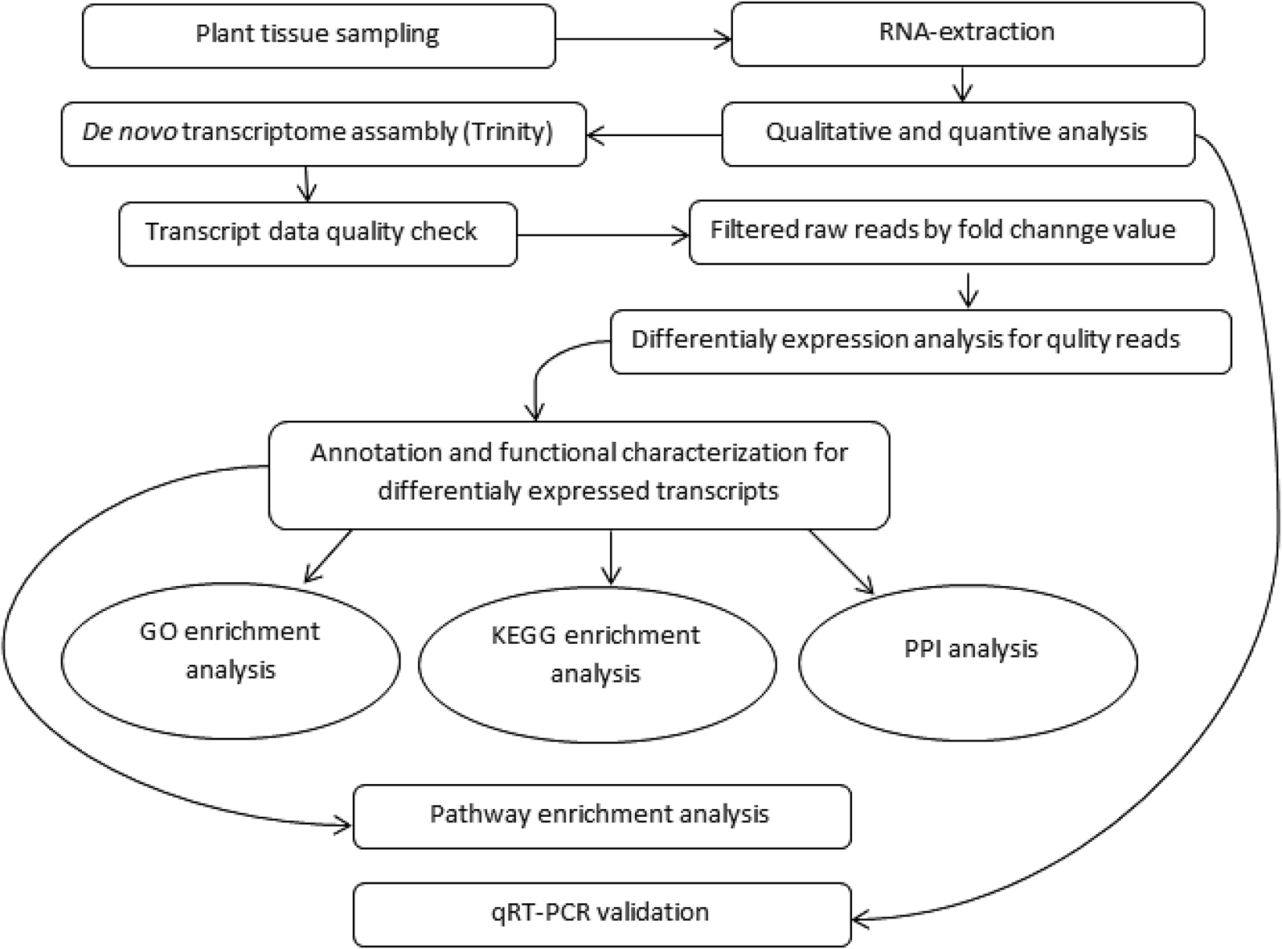
Work flow chart for transcriptomic profiling for crops

It has been observed that N uptake remains constant throughout domestication of extraordinary maize varieties but utilization of N enhanced, which support the hypothesis of conventional breeding programs improving NRE capacity [262]. Interestingly, inconsistency of overexpressed key enzymes (NR, NiR, GS, and GOGAT) for an improvement of NUE or phenotypic change is also a challenge [218, 231, 254, 262]. Due to these reasons, new molecular techniques like microarray and transcriptome (Fig. 6) are consider as emerging tools to study the response of plants whole genome.

The arrangement of known and unknown DNA samples on a solid support is known as microarray. Every microarray contains thousands of spots, each has less than 200 µM diameter and called probe [263]. These arrays may be in different formats and also probes can be smaller as oligonucleotides, cDNA or genomic sequences. Different techniques (photolithographic, nib, pin or inkjet) are employed to format. The probes are labelled radioactively or fluorescently and hybridization controlled electronically [264].

Whole genome sequencing is a modern approach to understand the changes at genomic level, expression level of genes and specific genes related to the desired traits. Good quality genome sequence information of ideotype rice and Arabidopsis plants are available for microarray analysis, but the transcriptomic profiling (Fig. 6) for whole genome sequencing of RNA is an excellent emerging technology for all plants [265, 266]. Molecular and physiological techniques have been employed in last two decades to know the differentially expressed genes (DEGs) in *Oryza sativa* [267, 268], *Sorghom bicolor* [269], *Glycin max* [270] and *Camilia sinensis* [271] for low nitrogen levels. Past studies mostly relied on single genotype for genes expression all over the world for low and normal nitrogen conditions either for nitrate or ammonium [267–271]. However, two genotypes of *Camilia sinensis* were studied and compared for both levels of nitrogen in ammonium form. Genotypic contrast for global genes expression and comparative analysis helped to compact the knowledge of candidate genes for NUE. A lot of information in literature regarding quantitative trait loci (QTLs) responding NUE are also available [272–274]. The combination of DEGs and QTLs datasets has great importance to develop new nitrogen use efficient genotypes in future [275].

Recent next generation sequencing technologies for transcriptomic profiling are helpful to understand the genes transcription and regulation of transcripts at all levels [276]. Illumina’s RNA-sequencing platform was used for transcriptomic exploration of genes expression to investigate the response of nitrogen nutritional stress in plants. It has been reported that the amino acid transporters in wheat plants play important role to transport nitrogen for development and a biotic stress conditions [277]. Based on the transcriptomic profiling Dai et al. studied the regulatory mechanism for storage protein in wheat grain in response to nitrogen supply during grain development [278]. Asparagine has crucial importance for nitrogen uptake in roots and considered as ideal nitrogen transporting molecule [258, 279, 280]. According to Curci et al. genes encoding asparagine were down regulated in leaves and roots of durum wheat under limited nitrogen [276]. It has been clearly observed that genes were down regulated in roots and leaves which were involved in carbon, nitrogen, amino acid metabolisms, and photosynthetic activity for plants grown under nitrogen free conditions [268].

## Conclusion

The agronomic and molecular approaches altogether have potential to improve nitrogen use efficiency. Nitrogen losses can be minimized by precision agriculture, cut off nitrogen dose, intercropping of legume and non-legume crops, improving plant populations and introducing nitrogen efficient genotypes. Although the studies have been conducted to improve nitrogen use efficiency of many crops by manipulating single or more genes but now the advanced technologies like whole genome sequencing are more important for future studies. Molecular breeding instead of conventional breeding is going to be more popular as of advancement in technologies. Wild genotypes are another option to improve NUE due to their more resistance against diseases, insect pest and have yield potential.

## Data Availability

Not applicable.

## Abbreviations

N: Nitrogen
NUE: Nitrogen use efficiency
NUtE: Nitrogen utilization efficiency
NU_p_E: Nitrogen uptake efficiency
aNUE: Agronomic nitrogen use efficiency
ATP: Adenosine triphosphate
PCR: Polymerase chain reaction
NRE: Nitrogen recovery efficiency
NO_3_^−^: Nitrate
NH_4_^+^: Ammonium
NR: Nitrate reductase
NiR: Nitrite reductase
GS: Glutamine synthetase
